# Ertapenem-induced neurotoxicity in an end-stage renal disease patient on intermittent haemodialysis: a case report

**DOI:** 10.1186/s12882-022-02980-8

**Published:** 2022-11-08

**Authors:** Shamira Shahar, Durga A. Arimuthu, Sadanah Aqashiah Mazlan

**Affiliations:** Nephrology Unit, Internal Medicine Department, Hospital Kajang, Selangor, Malaysia

**Keywords:** Case report, Ertapenem, Neurotoxicity, CKD 5D

## Abstract

**Background:**

Carbapenem-induced neurotoxicity is an unusual side effect, with seizure being the most commonly reported symptom. Among the carbapenems, imipenem-cilastin is classically associated with the most severe neurotoxicity side effects. Carbapenem is mainly excreted by the kidney and its half-life is significantly increased in patients with chronic kidney disease (CKD). Therefore, dose adjustment is necessary in such patients. Ertapenem-associated neurotoxicity is increasingly being reported in CKD patients, but rarely seen in patients with recommended dose adjustment.

**Case presentation:**

We report a case of a 56-year-old male patient with chronic kidney disease 5 on dialysis(CKD 5D). The patient presented with a history of fever, chills and rigours during a session of haemodialysis (HD). He was diagnosed with Enterobacter cloacae catheter-related blood stream infection and was started on ertapenem. After 13 days of ertapenem, he experienced an acute confusional state and progressed to having auditory and visual hallucinations. His blood investigations and imaging results revealed no other alternative diagnosis. Hence a diagnosis of ertapenem-induced neurotoxicity was made. He had complete resolution of symptoms after 10 days’ discontinuation of ertapenem.

**Conclusion:**

Our case draws attention to the risk of potentially serious toxicity of the central nervous system in HD patients who receive the current recommended dose of ertapenem. It also highlights that renal dosing in CKD 5D patients’ needs to be clinically studied to ensure antibiotic safety.

## Background

Carbapenems are a group of antibiotics with bactericidal activity towards both aerobic and anaerobic gram positive and negative organisms [1]. Ertapenem is a parenteral carbapenem with long-acting properties that is highly protein bound with a mean half-life of 3.8 to 4.4 hours [2]. Ertapenem is mainly renally excreted (80%) and one haemodialysis (HD) session can clear 30% of ertapenem dosage [3]. Carbapenem-induced neurotoxicity is an unusual side effect, With seizure being the most common symptom. Among the carbapenems, Imipenem-cilastin is classically associated with the most severe neurotoxicity symptoms. Recently, Ertapenem-associated neurotoxicity cases have been increasingly seen and reported [4, 5]. In a large retrospective study of ertapenem induced seizures, out of 1700 sampled patients 33 (1.9%) developed ertapenem-induced seizures, 2 (6%) Of whom were chronic kidney disease 5 on dialysis (CKD 5D) patients [6].

Here we reported a case of a CKD 5D patient who developed neurotoxicity while on recommended dose of ertapenem.

## Case presentation

We report a case of 52-year-old male patient with underlying CKD 5D on regular HD thrice a week for the past 1 year. Dialysis had been commenced via permanent catheter due to the patient being unsuitable for fistula creation. He is a teetotaller who had a normal cognitive baseline and denied any pre-existing psychiatric illness or head trauma. He also had a history of methicillin-susceptible Staphylococcus aureus (MSSA) catheter-related blood stream infection (CRBSI) 2 months prior to this presentation.

During a session of HD, he presented with a 3-day history of fever, chills and rigours. His blood investigation on admission was as followed: white cell counts 17.96 × 10^9/L, haemoglobin 8.3 g/dl, platelets 398 × 10^3/uL, urea 13.4 mmol/L, creatinine 788 umol/L, sodium 140 mmol/L, potassium 5.0 mmol/L, C-reactive protein 150 mg/L. He was empirically treated as MSSA CRBSI with intravenous (IV) cloxacillin and IV meropenem in view of his allergy to cephalosporin. On day 3 of admission, Enterobacter cloacae Group 1. Beta Lactamase (G1BL) was obtained from central and periphery blood culture. The antibiotic was changed to ertapenem 500 mg once daily after discussion with the hospital. Antimicrobial Stewardship Programme (AMS). On day 13 of IV ertapenem, he complained of visual and auditory hallucinations. He denied any fever or headaches. He was agitated due to the content of his hallucinations. His review of medications includes calcium carbonate 1 g thrice daily, calcitriol 0.25 mcg every other day, ferrous fumarate 200 mg once daily, perindopril 4 mg once daily.

On examination, he was not orientated to place and time. There were no signs of meningism and no neurological deficit, and other reviews of his system were unremarkable. Blood investigations at that point in time revealed the following: white cell count 7.96 × 10^9/L, haemoglobin 7.8 g/dl, platelets 471 × 10^3/uL, urea 16.4 mmol/L, creatinine 844 umol/L, sodium 140 mmol/L, potassium 4.2 mmol/L, chloride 98 mmol/L, magnesium 1.00 mmol/L, calcium 2.28 mmol/L, phosphorus 1.31 mmol/L, C-reactive protein 56 mg/L. Computed tomography imaging of the brain was normal. A lumbar puncture was not done as the patient had no fever or focal neurological signs.

He was started on risperidone to control his symptoms. At this point, the diagnosis of ertapenem-induced psychosis was made. Ertapenem was discontinued and gentamicin catheter lock was administered for 2 weeks. On day 10 after discontinuation, he had complete resolution of his symptoms. He was also weaned off risperidone uneventfully.

## Discussion and conclusion

Our case report highlights some important issues for consideration regarding the use of ertapenem in CKD 5D patients. In our case, the presentation of ertapenem-induced neurotoxicity manifested as visual and auditory hallucinations and agitation. The Naranjo Scale, which estimates the probability of an adverse drug reaction, was used to evaluate this potential causality. Our patient scored 6, which suggested probable causality [7]. Even though we were unable to obtain ertapenem drug levels during admission to assess the appropriateness of his dosing, the resolution of symptoms after discontinuation of ertapenem, and absence of other significant possible causes, indicated that it was high likely that his symptoms were due to ertapenem-induced encephalopathy. In clinical trials of ertapenem, the main neurological adverse events were headaches (2.2%) and seizures (0.2–0.5%) [8].

Hallucinations and acute confusional state, as seen in our case, have been described in other case reports [4, 5].

Ertapenem has high lipophilicity and its half-life is significantly increased in CKD patients especially those with low eGFR. Furthermore, only 30% of ertapenem is filtered during HD. Current dosing advise for dialysis patient were originated from old studies using low flux dialyser. High flux dialyser HD and HDF enhanced the clearance of drug as it able to remove larger molecule efficiently and may contribute to subtherapeutic level in CKD5D patient [9]. However, this might not be the reason in our case. Clearly there are other determinants that affected drug clearance in our patient.

Hypoalbuminemia, which is commonly seen in end-stage renal disease (ESRD), is a contributing factor that leads to increased free drug concentration and hence increases central nervous system (CNS) exposure. In few reviews of ertapenem-induced neurotoxicity in humans, the onset of symptoms varies from 4 to 10 days. However, patients with CKD 5D were found to have earlier onset (< 5 days) [4, 8].

In a case series study, in which ertapenem concentration was measured in one CKD 5D patient, it was found that 12 h post 500 mg IV ertapenem the plasma level at the beginning of HD was much higher than the minimum inhibitory concentration (MIC) 90 level. A further increase was seen before the second session of HD and the patient developed CNS symptoms during the fourth HD session (after seven consecutive doses) [3]. This contrasts with our patient, where the onset was later on day 13 of ertapenem. This difference in the onset of symptoms may be due to lack of awareness of the symptoms, and other contributing factors in our patient such as older age, low albumin and morbid obesity. In a retrospective study of ertapenem-induced seizures among CKD 5D patients, male sex, dementia and concomitant use of antibiotics were found to be the main predictors of seizure [1].

The current approved ertapenem dosing guidelines recommend an adjusted dose for CKD 5D patients. This dosage is based on pharmacokinetic data and has not been clinically studied. In a study on the AUC0-∞ of 1 g daily ertapenem in CKD 5D patients, it was found that the free drug concentration was five folds higher than in patients with mild renal impairment at eGFR > 30 [2]. This could be excessive as identified in our case and several other case reports [1, 10]. A recent pharmacokinetic study of the effects of ertapenem when administered as a 500 mg dose three times weekly found that a plasma concentration above 2 mg/L was maintained (selected based on the MIC90 and the CLSI/FDA susceptibility breakpoint) [11].

This finding is exciting and could be of practical benefit for CKD 5D patients, where a three times weekly dose could be administered via a HD line, thus conserving other vessels for future HD access.

This case draws attention to the risk of potentially serious CNS toxicity in HD patients who receive the current recommended dose of ertapenem. Increased awareness, prompt diagnosis and immediate discontinuation are all crucial to avoid unnecessary investigations and potentially appalling complications. Importantly, this report highlights that renal dosing in CKD 5D patients’ needs to be studied clinically and pharmacokinetically to ensure the antibiotic safety.

## Data Availability

Not applicable.

## Abbreviations

HD: Haemodialysis
HDF: Haemodialysis filtration
CKD 5D: Chronic kidney disease 5 on dialysis
eGFR: Estimated glomerular filtration rate
MSSA: Methicillin susceptible Staphylococcus Aureus
CRBSI: Catheter-related blood stream infection
AMS: Antimicrobial stewardship programme
WCC: White cell count
Hb: Haemoglobin
CNS: Central nervous system
ESRD: End-stage renal disease
MIC: Minimum inhibitory concentration
FDA: Food and Drug Administration
CLSI: Clinical Laboratory Standard Institute

## References

[1] El Nekidy WS, Elrefaei H, St. John TJL, Attallah NM, Kablaoui F, Nusair A (2021). Neurotoxicity in Hemodialysis Patients—Safe and Effective Dosing Is Still Needed: A Retrospective Study and Literature Review. Ann Pharmacother.

[2] Mistry GC, Majumdar AK, Swan S, Sica D, Fisher A, Xu Y (2006). Pharmacokinetics of ertapenem in patients with varying degrees of renal insufficiency and in patients on hemodialysis. J Clin Pharmacol.

[3] Lee KH, Ueng YF, Wu CW, Chou YC, Ng YY, Yang WC (2015). The recommended dose of ertapenem poses a potential risk for central nervous system toxicity in haemodialysis patients - Case reports and literature reviews. J Clin Pharm Ther.

[4] Veillette JJ, Van Epps P. Ertapenem-induced hallucinations and delirium in an elderly patient. Consultant Pharmacist. Am Soc Consult Pharm. 2016;30:207–14. 10.4140/TCP.n.2016.207 27056357

[5] Adams R, Chopra P, Miranda R, Calderon A (2020). Ertapenem-induced encephalopathy BMJ Case Rep.

[6] Lee YC, Huang YJ, Hung MC, Hung SC, Hsiao CY, Cho HL, et al. Risk factors associated with the development of seizures among adult patients treated with ertapenem: a matched case-control study. PLoS One. 2017;12(7):e0182046. 10.1371/journal.pone.0182046 PMC5536326 28759588

[7] Naranjo CA, Busto U, Sellers EM, Sandor P, Ruiz I, Roberts EA (1981). A method for estimating the probability of adverse drug reactions. Clin Pharmacol Ther.

[8] Deshayes S, Coquerel A, Verdon R. Neurological adverse effects attributable to β-lactam antibiotics: a literature review. Drug Safety. Springer Int Publishing. 2017;40:1171–98. 10.1007/s40264-017-0578-2 28755095

[9] Jager NGL, Zandvliet AS, Touw DJ, Penne EL (2017). Optimization of anti-infective dosing regimens during online haemodiafiltration. Clin Kidney J.

[10] Wen MJ, Sung CC, Chau T, Lin SH (2013). Acute prolonged neurotoxicity associated with recommended doses of ertapenem in 2 patients with advanced renal failure. Clin Nephrol.

[11] Ueng Y-F, Wang H-J, Wu S-C, Ng Y-Y (2019). A Thrice-Weekly Ertapenem Regimen Is Practical for Hemodialysis Patients. Antimicrobs Agent Chemother.

